# Brain Histology and Immunohistochemistry After Resuscitation From Hemorrhagic Shock in Swine With Pre-Existing Atherosclerosis and Sodium Thiosulfate (Na_2_S_2_O_3_) Treatment

**DOI:** 10.3389/fmed.2022.925433

**Published:** 2022-06-30

**Authors:** Nicole Denoix, Oscar McCook, Angelika Scheuerle, Thomas Kapapa, Andrea Hoffmann, Harald Gündel, Christiane Waller, Csaba Szabo, Peter Radermacher, Tamara Merz

**Affiliations:** ^1^Clinic for Psychosomatic Medicine and Psychotherapy, Ulm University Medical Center, Ulm, Germany; ^2^Institute for Anesthesiological Pathophysiology and Process Engineering, Ulm University Medical Center, Ulm, Germany; ^3^Division of Neuropathology, Institute for Pathology, Ulm University Medical Center, Ulm, Germany; ^4^Clinic for Neurosurgery, Ulm University Medical Center, Ulm, Germany; ^5^Department of Psychosomatic Medicine and Psychotherapy, Nuremberg General Hospital, Paracelsus Medical University, Nuremberg, Germany; ^6^Department of Science and Medicine, University of Fribourg, Fribourg, Switzerland

**Keywords:** hydrogen sulfide, cystathionine-γ-lyase, cystathionine-β-synthase, oxytocin receptor, hypoxia, glial fibrillary acidic protein, nitrotyrosine, paraventricular nucleus

## Abstract

**Background:**

The hydrogen sulfide (H_2_S) and the oxytocin/oxytocin receptor (OT/OTR) systems interact in the central nervous and cardiovascular system. As a consequence of osmotic balance stress, H_2_S stimulates OT release from the paraventricular nuclei (PVN) in the hypothalamic regulation of blood volume and pressure. Hemorrhagic shock (HS) represents one of the most pronounced acute changes in blood volume, which, moreover, may cause at least transient brain tissue hypoxia. Atherosclerosis is associated with reduced vascular expression of the main endogenous H_2_S producing enzyme cystathionine-γ-lyase (CSE), and, hence, exogenous H_2_S administration could be beneficial in these patients, in particular after HS. However, so far cerebral effects of systemic H_2_S administration are poorly understood. Having previously shown lung-protective effects of therapeutic Na_2_S_2_O_3_ administration in a clinically relevant, long-term, porcine model of HS and resuscitation we evaluated if these protective effects were extended to the brain.

**Methods:**

In this study, available unanalyzed paraffin embedded brain sections (Na_2_S_2_O_3_ *N* = 8 or vehicle *N* = 5) of our recently published HS study were analyzed *via* neuro-histopathology and immunohistochemistry for the endogenous H_2_S producing enzymes, OT, OTR, and markers for brain injury and oxidative stress (glial fibrillary acidic protein (GFAP) and nitrotyrosine).

**Results:**

Neuro-histopathological analysis revealed uninjured brain tissue with minor white matter edema. Protein quantification in the hypothalamic PVN showed no significant inter-group differences between vehicle or Na_2_S_2_O_3_ treatment.

**Conclusions:**

The endogenous H_2_S enzymes, OT/OTR co-localized in magnocellular neurons in the hypothalamus, which may reflect their interaction in response to HS-induced hypovolemia. The preserved blood brain barrier (BBB) may have resulted in impermeability for Na_2_S_2_O_3_ and no inter-group differences in the PVN. Nonetheless, our results do not preclude that Na_2_S_2_O_3_ could have a therapeutic benefit in the brain in an injury that disrupts the BBB, e.g., traumatic brain injury (TBI) or acute subdural hematoma (ASDH).

## Introduction

Recently, we described lung-protective effects of therapeutic sodium thiosulfate (Na_2_S_2_O_3_) administration in a clinically relevant, long-term, porcine model of hemorrhagic shock (HS) and resuscitation in swine with pre-existing atherosclerosis (1). The lung-protective effects comprised amelioration of shock-induced impairment of lung mechanics and gas exchange, and coincided with a significantly higher glucocorticoid receptor (GR) expression in the lung tissue of treated animals (1). It is not yet understood, to what extent relevant effects of systemic Na_2_S_2_O_3_ administration reach the brain (2–4). However, exogenous hydrogen sulfide (H_2_S) administration was shown to stimulate H_2_S availability by upregulation of its endogenous enzymes cystathionine-γ-lyase (CSE) and cystathionine-β-synthase (CBS) (5, 6) as an adaptive stress response (7). In a previous study, we reported a significant reduction of CSE in the media of the coronary arteries in atherosclerotic pigs, which was further aggravated in circulatory shock (8). A reduction of CSE is implicated in barrier dysfunction in the kidney (9, 10) and reduced vascular H_2_S availability (6). Therefore, Na_2_S_2_O_3_ administration after HS may be beneficial for atherosclerotic patients since the lack of vascular H_2_S production might be compensated by the exogenous administration. Moreover, H_2_S and the oxytocin (OT)/ oxytocin receptor (OTR) systems interact in trauma in the heart and the brain (11–15) and are both reported to have anti-oxidative, and vasculo-protective effects (15). Hence, activation of either system could be beneficial in HS, since severe hypovolemia can induce at least transitory cerebral tissue hypoxia events (16–19), redox imbalance (20), barrier dysfunction (18, 21) and, upon resuscitation, ischemia/reperfusion (I/R) injury (22).

Interestingly, HS has been shown to induce OT release (23), and OTR upregulation was associated with protection against ischemic injury around the cerebral vasculature and was co-localized with glial fibrillary acidic protein (GFAP), an established marker of reactive astroglial cells, which are reported to be increased in regions of ischemic injury. Moreover, H_2_S can stimulate hypothalamic release of OT in response to fluid deprivation in rats and osmotic challenge in uninjured rat hypothalamic explants, respectively (23, 24). Central to these observations is the paraventricular nucleus (PVN) (25) of the hypothalamus, the PVN is highly vascularized, protected by the BBB and adjacent to the third ventricle, (not to be confused with periventricular nucleus which lies below the third ventricle and does not have an effective BBB), as part of the hypothalamic-pituitary-adrenal axis (26), important interface in the regulation of fluid homeostasis (27) and site for cardiorespiratory output, blood pressure and heart rate regulation during hypoxia (23, 28). Thiosulfate can release H_2_S under hypoxic conditions, thus we investigated Na_2_S_2_O_3_ as a H_2_S donor during resuscitated HS, since it is a clinically approved drug with a good safety profile and antioxidant properties (29–31).

Thiosulfate is proposed to be “a circulating ‘carrier’ molecule of beneficial effects of H_2_S” (4, 30). Furthermore, Na_2_S_2_O_3_ elicited neuroprotective effects in a rodent global cerebral ischemia model (4) and its therapeutic efficacy has been shown in other *in vivo* models of organ failure and cardiovascular dysfunction (31, 32). We therefore investigated the effects of Na_2_S_2_O_3_ on the brain, and how the H_2_S and OT/OTR systems in swine with pre-existing atherosclerosis are regulated after HS and resuscitation. The hypothalamic PVN was investigated, since H_2_S and OT are known to interact in the hypothalamic regulation of fluid homeostasis and HS represents the most drastic shift in blood volume after trauma. The data presented are a post hoc analysis of material available from the above-mentioned previous study (1).

## Materials and Methods

The present study is a *post-hoc* analysis of post mortem brain tissue sections obtained from the previous study of Datzmann et al. (1). Experiments were conducted after approval by the Federal Authorities for Animal Research (Regierungspräsidium Tübingen; Reg.-Nr. 1341, date of approval May 2, 2017), and the local University of Ulm Animal Care Committee, in adherence to the National Institute of Health Guidelines on the Use of Laboratory Animals and the European Union “Directive 2010/63/EU on the protection of animals used for scientific purposes.” Anesthesia, surgical instrumentation, as well as the protocol of hemorrhage and resuscitation have been described in detail previously (1). Briefly, induction of HS was performed by passive blood withdrawal of 30% of the calculated total blood volume. Hemorrhage was titrated to a targeted mean arterial pressure of 40 ± 5 mmHg for 3 h. Resuscitation of the animals included shed blood re-transfusion, fluid administration (balanced electrolyte solutions), vasopressor support (norepinephrine) based on need to restore mean arterial pressure to baseline values, and lung-protective mechanical ventilation. Sodium thiosulfate (Na_2_S_2_O_3_) (Dr. Franz Köhler Chemie GmbH, Bensheim, Germany, 25%, diluted in 500 mL NaCl 0.9%; infusion rate 0.1 g·kg^−1^·h^−1^) (*N* = 8) or the vehicle solution (2 mL·kg^−1^·h^−1^) (*N* = 5) was administered during the first 24 h of re-transfusion. After 72 h of intensive care treatment or premature termination of the experiment due to pre-defined termination criteria (acute respiratory distress syndrome, refractory hypotension), pigs were sacrificed under further deepened anesthesia, by injection of potassium chloride, and the brain was immediately removed (1).

### Experimental Protocol

Available porcine brain tissue sections from a recently published HS study (1) were analyzed in this study. Brain sections from seven female and six castrated male “Familial Hypercholesterolemia Bretoncelles Meishan” (FBM) pigs with median (interquartile range) body weight of 58 (49, 66) kg and age of 24 (23, 28) months, were investigated. These FBM pigs are a model for “human-like coronary atherosclerosis” (8, 33, 34). Group assignment was performed randomly, irrespective of sex.

### Neuro-Histophathology and Immunohistochemistry

All brains were identically fixed in 4% formalin for 6days, dissected into 4 mm thick consecutive coronal sections: frontal to occipital. If the macroscopic section was too large to fit as a whole into the embedding cassette (26 x 31 x 4 mm) they were laid flat and further dissected in up to five pieces in a manner which allows for reconstruction of the entire section. For the purpose of this study the macroscopic section, which included the hypothalamus was selected for analysis. The tissue was then dehydrated and embedded in paraffin blocks. 3–5 μm sections, were deparaffinized in xylene, and rehydrated in a graded series of ethanol and deionized water. Hematoxylin and eosin (HE) staining was performed for general neuro-histophathological evaluation, an experienced neuro-histopathologist (AS) focused on determining if hypoxic events led to brain tissue injury in cortico-subcortical brain regions. Criteria to evaluate brain tissue injury included: morphology of nerve and glia cells, white matter and perivascular edema formation, necrosis of parenchyma and appearance of eosinophilic cells. Due to minor injury, descriptive evaluation was performed without quantification.

Immunohistochemical (IHC) analysis with a colorimetric detection system, as method of choice, allowed for visualizing tissue architecture, cellular morphology and cytoplasmic or nuclear protein localization, in the general landscape of the brain. Important here, by not homogenizing the tissue spatial expression patterns remain intact, and it is possible to distinguish e.g,. between vasculature (containing blood) and brain parenchyma, which is a known confounding factor in techniques requiring tissue homogenates.

IHC was performed as previously described (15). Briefly, after deparaffinizing, heat-induced antigen retrieval in citrate buffer (pH 6) was performed, followed by blocking with normal goat serum (10%) before incubating with the following primary antibodies: endogenous H_2_S producing enzymes anti-cystathionine-γ-lyase (CSE) (Protein Tech, 12217-1-AP, RRID:AB_2087497) and anti-cystathionine-β-synthase (CBS) (Protein Tech, 14787-1-AP, RRID:AB_2070970), anti-OT (Millipore, AB911, RRID:AB_2157629), anti-OTR (Protein Tech, 23045-1-AP, RRID:AB_2827425), which were verified in porcine cerebral tissue previously by Denoix et al. (22), anti-GFAP (Abcam, ab7260, RRID:AB_305808) as a marker of cerebral injury, anti-pig Albumin (Abcam, ab79960, RRID:AB_1658916) as a marker of barrier dysfunction and anti-nitrotyrosine (Merck Millipore, ab5411, RRID:AB_177459), as a marker of oxidative and nitrosative stress, since NO can react with superoxide to peroxynitrite, leading to nitrotyrosine formation due to nitration of protein tyrosine residues (13). All primary antibodies were titrated to their optimal dilution according to the manufacturer recommendations (see Table 1). Negative controls were performed concurrently (15) by incubation with diluent instead of primary antibody in order to control for specificity of the secondary system, or, whenever available, we performed pre-incubation of the primary antibody with the respective immunogen peptide and incubated the brain tissue with the pre-absorbed primary antibody instead of normal primary antibody (15). Additionally, NCBI BLAST searches were performed in order to compare immunogen sequences of the used antibodies to the sus scrofa database (courtesy of the U.S. National Library of Medicine) (Table 1) to further confirm the specificity of the used primary antibodies.

**Table 1:** Primary antibodies (anti-human) to sus scrofa Protein BLAST search.

**Primary antibody (source, catalog No., RRID)**	**Host species**	**Query cover**	**Homology**	**Immunogen sequence**	**Concentration used for IHC**
**anti-CSE** (Protein Tech, 12217-1-AP, AB_2087497)	Rabbit Polyclonal	100 %	87.05 %	Gamma cystathionase fusion protein Ag2872	1:200
**anti-CBS** (Protein Tech, 14787-1-AP, AB_2070970)	Rabbit Polyclonal	100 %	90.46 %	CBS fusion protein Ag6437	1:100
**anti-OT** (Millipore, AB911, AB_2157629)	Rabbit Polyclonal	100 %	100 %	CYIQNCPLG (Synthetic oxytocin (Sigma) conjugated to thyroglobulin)	1:500
**anti-OTR** (Protein Tech, 23045-1-AP, AB_2827425)	Rabbit Polyclonal	92 %	83.05 %	Oxytocin Receptor fusion protein Ag19074	1:100
**anti-GFAP** (Abcam, ab7260, AB_305808)	Rabbit Polyclonal	100 %	92.13 %	full length GFAP sequence UniProt ID: 14136	1:2500

Primary antibodies were detected via the Dako REAL detection system based on alkaline phosphatase conjugated secondary antibodies (anti-mouse; anti-rabbit) and visualized with a Fast Red-type chromogen followed by counterstaining with Mayer's hematoxylin (9, 10). A Zeiss Axio Imager A1 microscope with a 10X objective was used for visualizing the slides. Multiple 800,000-μm^2^ sections were used for quantification of % positive area using the Zen Image Analysis Software (Zeiss). Data are presented as % positive area.

### Statistical Analysis

Graph Pad Prism Version 8 was used for statistical analysis. Normal distribution was excluded with the Kolmogorov–Smirnov test and inter-group differences were analyzed with the Mann-Whitney rank sum test. The data are presented as median (quartiles) with interquartile ranges.

## Results

### Minor Neuro-Histopathological Damage

**Figure 1:**
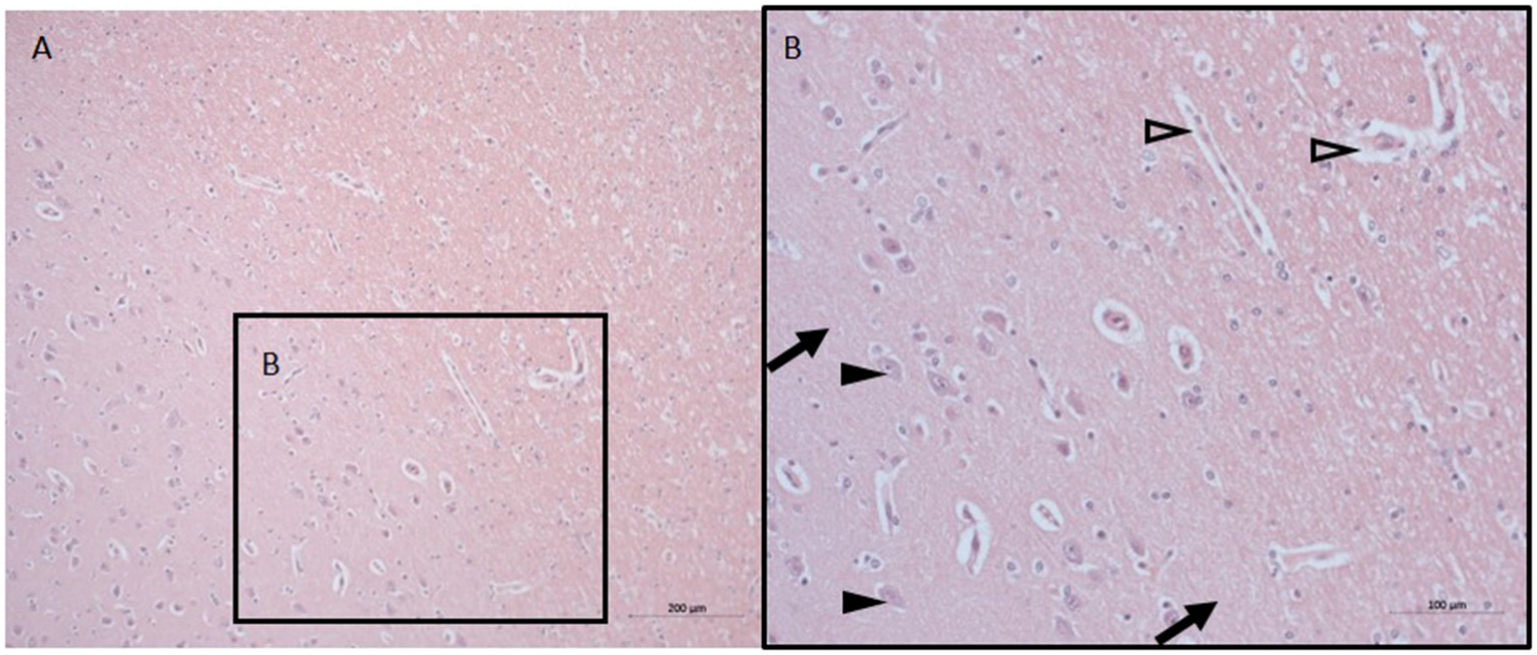
Neuro-histopathological evaluation. Exemplary pictures of HE staining in cortico-subcortical brain tissue with signs of minor perivascular edema in sections from the same macroscopic sectional plane as the PVN at 10X **(A)** and 20X **(B)**. Black arrows point to intact parenchyma, black arrowheads depict nerve cells, open arrowheads point to minor perivascular edema. HE, Hematoxylin and eosin; PVN, paraventricular nucleus.

The histopathological findings were minimal with signs of discrete swelling and minor perivascular edema in cortico-subcortical brain regions (in the same macroscopic sectional plane as the PVN, see Figure 1), otherwise the brain tissue looked histologically uninjured, presenting with normal appearing singular heterotopic ganglion cells, and intact parenchyma. There were no indications of blood brain barrier disruption.

### Lack of Effect of Na_2_S_2_O_3_ on CSE, CBS, OT, OTR, GFAP and Nitrotyrosine in the PVN

**Figure 2:**
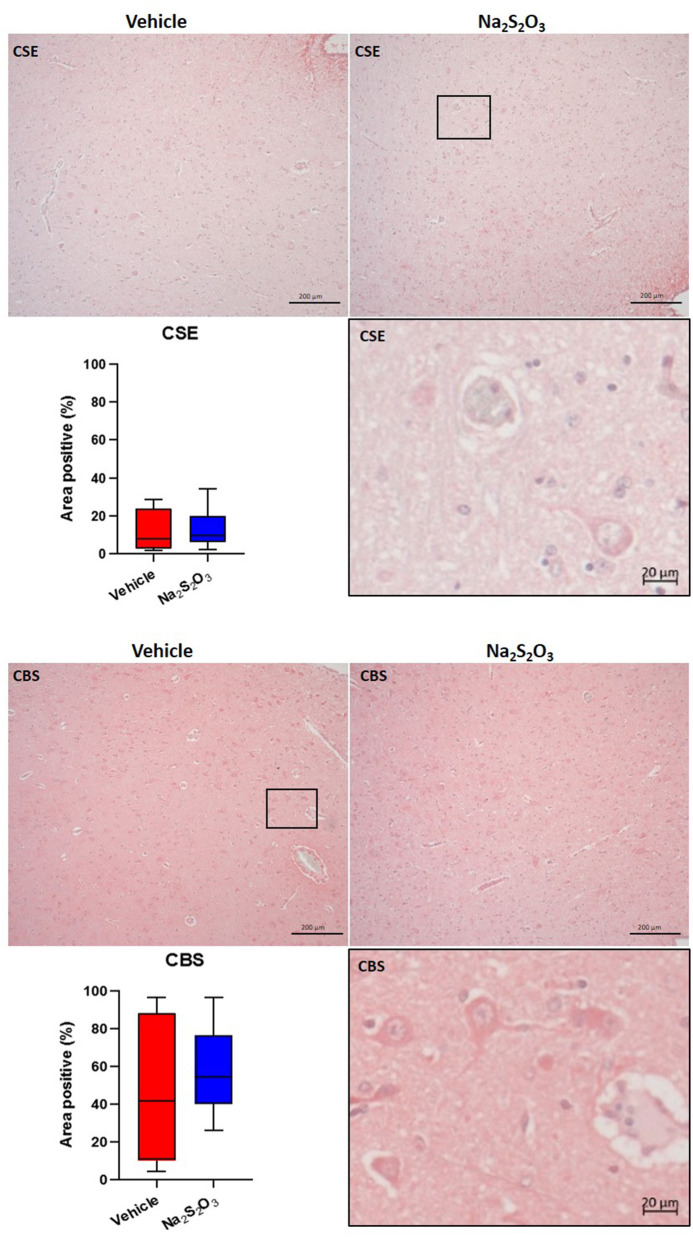
H_2_S producing enzymes in the porcine PVN. Quantification of the immunohistochemical stainings as positive percentage area in the PVN for CSE (vehicle: *n* = 4; Na_2_S_2_O_3_: *n* = 6) and CBS (vehicle: *n* = 5; Na_2_S_2_O_3_: *n* = 7). Boxes represent the interquartile ranges with the median (black line), whiskers represent minimum and maximum values. Exemplary immunohistochemical pictures of CSE and CBS in the PVN of vehicle and Na_2_S_2_O_3_ treated animals (10X). Higher magnification pictures of CSE and CBS originate from the black box in the respective 10X picture. PVN, paraventricular nucleus; CSE, cystathionine-γ-lyase; CBS, cystathionine-β-synthase; Na_2_S_2_O_3_, sodium thiosulfate.

**Figure 3:**
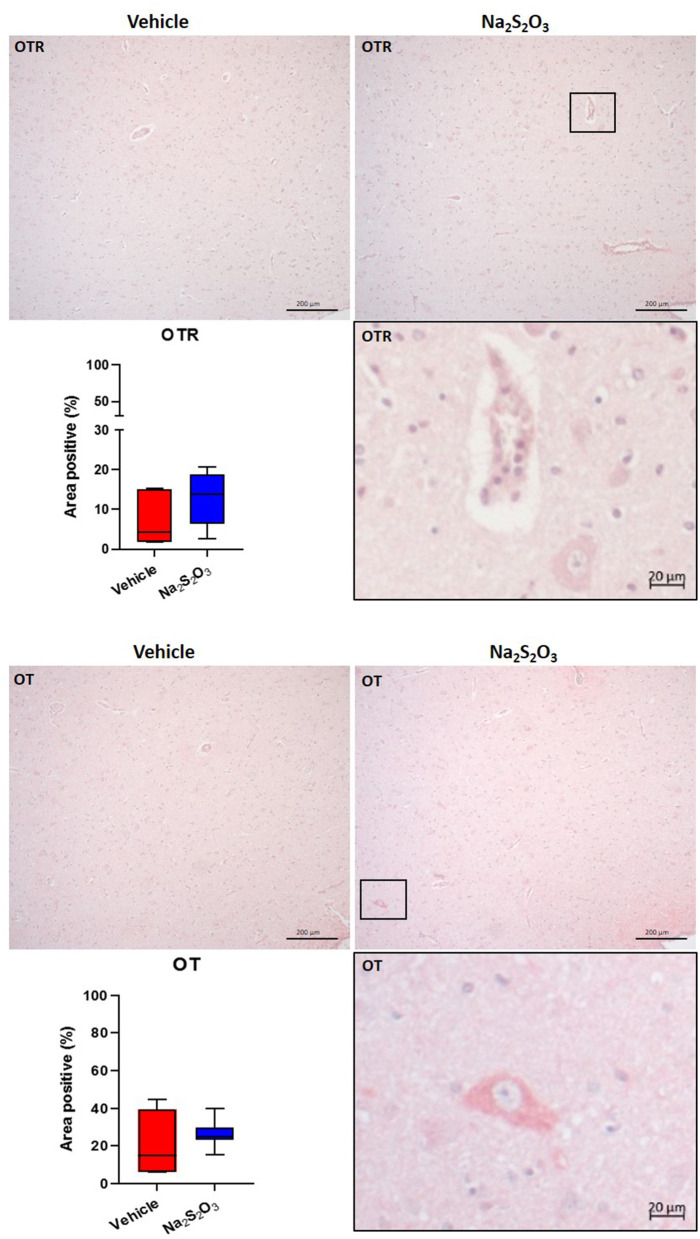
OTR and OT in the porcine PVN. Quantification of the immunohistochemical stainings as positive percentage area in the PVN for the OTR (vehicle: *n* = 5; Na_2_S_2_O_3_: *n* = 7) and OT (vehicle: *n* = 5; Na_2_S_2_O_3_: *n* = 7). Boxes represent the interquartile ranges with the median (black line), whiskers represent minimum and maximum values. Exemplary immunohistochemical pictures of the OTR and OT in the PVN of vehicle and Na_2_S_2_O_3_ treated animals (10X). Higher magnification pictures of the OTR and OT originate from the black box in the respective 10X picture. OT, oxytocin; OTR, oxytocin receptor; Na_2_S_2_O_3_, sodium thiosulfate.

**Figure 4:**
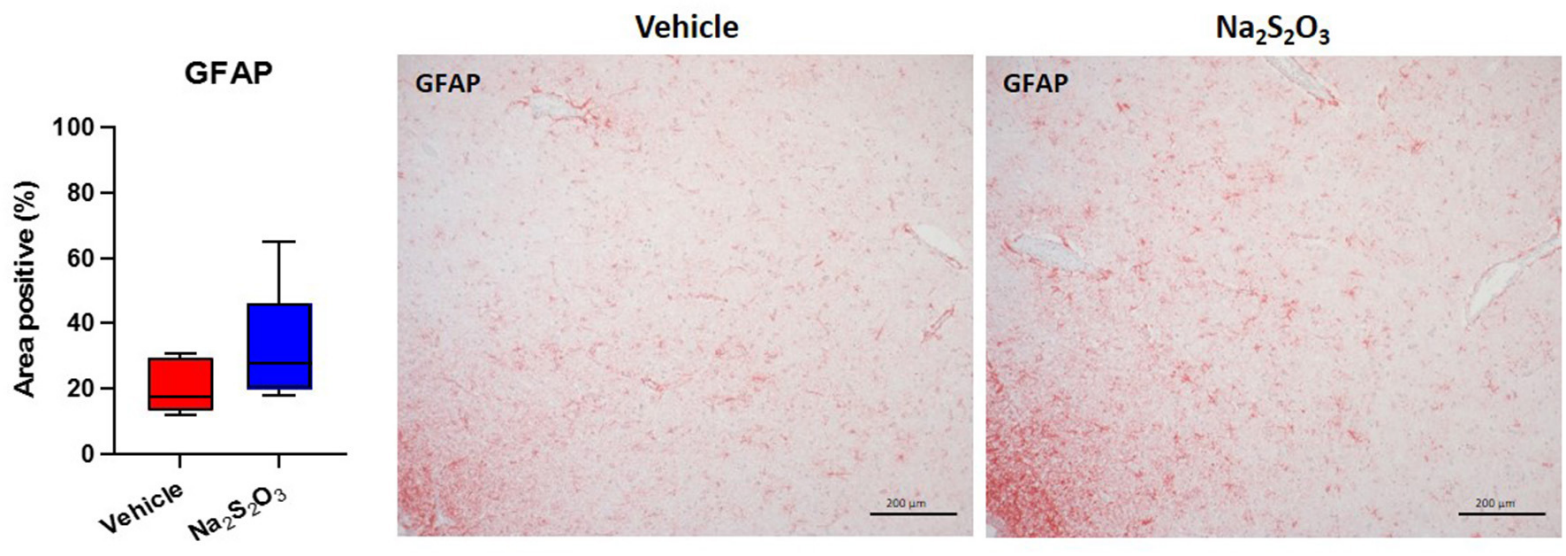
GFAP in the porcine PVN. Quantification of the immunohistochemical stainings as positive percentage area in the PVN for GFAP (vehicle: *n* = 5; Na_2_S_2_O_3_: *n* = 8). Boxes represent the interquartile ranges with the median (black line), whiskers represent minimum and maximum values. Exemplary immunohistochemical pictures of GFAP in the PVN of vehicle and Na_2_S_2_O_3_ treated animals (10X). PVN, paraventricular nucleus; GFAP, glial fibrillary acidic protein; Na_2_S_2_O_3_, sodium thiosulfate.

None of the proteins of interest showed any significant inter-group difference in the porcine hypothalamic PVN (Figures 2–4). The endogenous H_2_S producing enzymes CSE, CBS, and OT and its receptor were present in the porcine hypothalamic PVN (Figures 2, 3). CSE, CBS, OT and the OTR were found to be present in the magnocellular neurons of the PVN in consecutive sections of the porcine hypothalamus (35). GFAP was also present in the PVN of both groups (see Figure 4) but, again, did not show a significant inter-group difference. Nitrotyrosine showed negligible to no expression in the analyzed tissue specimens (vehicle: *n* = 5, 0.00[0.00; 0.00] %; Na_2_S_2_O_3_: *n* = 8, 0.01[0.00; 0.03] %). No Albumin extravasation could be detected (data not shown).

## Discussion

The aim of this study was to investigate the protein levels of CSE, CBS, OT, and OTR in the PVN after HS-and resuscitation in a clinically relevant, long-term porcine model, since HS represents one of the most pronounced acute fluid shifts in blood volume. Given the fact that the H_2_S and OT systems interact in trauma (11–15) and in hypothalamic regulation of fluid homeostasis (23–28), we hypothesized that Na_2_S_2_O_3_ would lead to changes in the expression of theses markers. The results in this porcine model of HS-and resuscitation, showed mostly uninjured brain tissue with minor perivascular edema in cortico-subcortical brain regions. Immunohistochemical data revealed that (i) the proteins of interest CSE, CBS, OT and OTR were all localized in the porcine PVN after HS, but in contrast to our underlying hypothesis, (ii) there were no significant differences in protein levels of CSE, CBS, OT, OTR, GFAP nor nitrotyrosine in the PVN of either group after HS.

Previously, we showed in atherosclerotic swine that CSE, CBS, OT and the OTR were found to be present in magnocellular neurons in consecutive sections of the porcine hypothalamus after HS, which may be indicative of their hypothalamic interaction in response to HS-induced hypovolemia (35). In rodents, there is evidence that Na_2_S_2_O_3_ can reach the brain 90 min after intraperitoneal administration, as demonstrated by elevated Na_2_S_2_O_3_ levels in plasma and brain tissue, mediating protective effects after neuronal I/R injury in un-resuscitated, young and otherwise healthy male mice (4). In our model of HS, we did not see the same results as in the previously mentioned I/R injury rodent model, which may be due to the fact that our large animal model included a pre-existing comorbidity and was resuscitated in an ICU, thus mimicking the human situation unlike the rodent model (4). In fact, the pig is a very relevant translational model: structurally very similar to the human brain, the presence of gyri and sulci (gyrencephalic brain), white matter to gray matter proportion and tentorium cerebelli, unlike in the rodent brain, is reflective of the human pathophysiology (36). Administration of Na_2_S_2_O_3_ after brain injury in rodents stimulated H_2_S availability by upregulation of CBS (5). In contrast, in this pig study, CSE, CBS and OT/OTR expression levels did not show any inter-group difference. Although the systemic blood sulfide levels were significantly higher in animals at 24 h of Na_2_S_2_O_3_ administration compared to vehicle treated swine (1). Clearly, Na_2_S_2_O_3_ administration had lung-protective effects and induced higher pulmonary GR expression, whereas the other visceral organs and blood cells were not affected (1). Organ specific pathophysiology has been shown in other models of circulatory shock, which led to the concept of compartmentalization, suggesting that there are differences in response patterns between single organs and systemic factors, as proposed by Cavallion et al. (37). The compartmentalization in the brain may be due in part to the blood brain barrier (BBB). The lack of effect of Na_2_S_2_O_3_ on the evaluated parameters in the brain in our pig model may suggest its inability to cross the BBB, which is, in contrast to the aforementioned results in rodents, but would be, however, consistent with findings in humans that show that Na_2_S_2_O_3_ does not cross the intact BBB (3, 38). In comparison to our applied dose of Na_2_S_2_O_3_ (0.1 g·kg^−1^·h^−1^), even more than 5-fold higher doses of Na_2_S_2_O_3_ did not cross the intact BBB in humans (3, 38). The lack of resuscitation in the reported rodent studies might have facilitated the breakdown of the BBB and thus allowed for Na_2_S_2_O_3_ to cross the dysfunctional barrier and promote neuroprotective effects. These results, however, would not translate to the human clinical situation, which would always entail resuscitation to defined neuro-intensive care endpoints. The clinical scenario was closely replicated in the present large animal study, suggesting that the results presented here are very relevant observation for translational medicine. Based on previous data from rodent experiments, which show that exogenous H_2_S administration can induce neuroprotective effects and the hypothalamic OT system and endogenous H_2_S producing enzymes in response to fluid shifts (5, 23, 24, 27), we specifically looked for CSE, CBS, OT and OTR in the PVN with other markers, relevant to hypoxia, fluid shifts and injury (GFAP, Albumin and nitrotyrosine) after resuscitated HS with and without systemic Na_2_S_2_O_3_ administration and could not find significant effects. Our negative findings, though disappointing, assume significance in the light of the many successful unresuscitated rodent pre-clinical trials that ultimately failed to translate to the clinic in neurotrauma research (15, 36).

It remains to be elucidated whether the Na_2_S_2_O_3_-mediated neuroprotective effects (observed *in vitro* and in *in vivo* experiments in young and otherwise healthy male mice) as well as the stimulating effect of H_2_S on OT release within the hypothalamus during osmotic balance stress (observed in healthy, fluid deprived rats or uninjured, brain explants osmotically challenged), are translatable into large animals or humans (4, 23, 24). Long-term studies in large animals, which focus on the brain after HS, are scarce and report impaired cerebral microcirculation and perfusion pressure, increase in cerebrovascular resistance, altered energy metabolism and BBB dysfunction due to damaged astroglial cells immediately after blood loss (18, 39). GFAP expression is reported to increase in ischemic events, but there was no difference in GFAP expression between vehicle and Na_2_S_2_O_3_ treated animals after HS in the current study. To the best of our knowledge, reports on HS-induced histopathological changes or protein expression variations after profound tissue hypoxia in adult porcine brain tissue are lacking. Most large animal studies, either perform a combination of HS and acute brain injury or cardiac arrest, which indicates a “no-flow” instead of a “low-flow” state and are therefore not comparable to our results (40, 41). Large animal studies that specifically investigate cerebral effects of HS often lack post HS intensive care (42). Although it has been shown for both pigs and rodents that implementation of resuscitative measures effectively preserves the BBB (43), which was also the case in our study, as indicated by the lack of albumin extravasation. The existing literature about HS alone without brain injury does not seem to result in major morphological damage (42), which is in agreement with our findings. Histopathological examination of brain sections in this study revealed generally uninjured brain tissue morphology with only minor perivascular edema in cortico-subcortical brain regions.

Hypoxia-induced cellular injury can start immediately with hemodynamic decompensation (18, 20) and may cause damage that is first visible on protein level. We recently characterized the gyrencephalic porcine brain after acute subdural hematoma with concomitant pressure-induced brain injury and BBB disruption (15). CBS, OT and the OTR were more localized at the injury site, whereas CSE was constitutively expressed in the uninjured brain tissue and reduced at the injury site (15). In the present study we now quantified protein levels in the PVN after a massive drop in blood volume, which revealed that OT and the OTR are present in the porcine PVN, and that CBS is generally expressed in higher quantity than CSE.

### Limitations of the Study

The present study was a *post-hoc* analysis of brain tissue from a previous experiment. The brain was not a target organ to be investigated in the original study. Thus, no brain monitoring was performed and we cannot comment on how the cerebral perfusion pressure and/or cerebral blood flow have been affected by the hemorrhagic shock. It may well-be, that cerebral autoregulation prevented any effect of the lower MAP on cerebral perfusion.

## Conclusion

We found neither neuro-histopathological differences, nor differences in the pattern or levels of CSE, CBS, OT, OTR, GFAP nor nitrotyrosine in the porcine PVN between Na_2_S_2_O_3_ and vehicle administration after HS-and resuscitation. These findings are in contrast to the findings in unresuscitated rodents and may be indicative of the reason the neurotrauma pre-clinical trials in rodents have failed to translate to the clinic. The presence of CSE, CBS, OTR and OT in magnocellular neurons of the porcine hypothalamus may reflect their interaction in response to HS-induced hypovolemia (35). The lack of inter-group differences in the PVN may be attributed to the organ-specific compartmentalization due to preserved impermeability of the intact BBB for Na_2_S_2_O_3_ as observed in humans. Nonetheless, our results do not preclude that Na_2_S_2_O_3_ could have a therapeutic benefit in the brain in an injury that disrupts the BBB, e.g., traumatic brain injury (TBI), acute subdural hematoma (ASDH), asphyxiation or “no flow” scenarios such as cardiopulmonary resuscitation.

## Data Availability Statement

The raw data supporting the conclusions of this article will be made available by the authors, without undue reservation.

## Ethics Statement

The animal study was reviewed and approved by Regierungspräsidium Tübingen; Reg.-Nr. 1341, date of approval May 2, 2017.

## Author Contributions

ND performed the immunohistochemistry, data analysis and interpretation, and drafting of the manuscript. AH performed animal experiments and removal of the brain during the necropsy. AS performed the neuro-histopathological evaluation with ND and helped with data interpretation, critical comments, and expert feedback on the manuscript. CS, CW, HG, and TK contributed critical comments and expert feedback on the manuscript. OM and TM contributed to the experimental design, supervising immunohistochemistry, data interpretation, and writing of the manuscript. PR contributed to the study design and edited and approved the final version of the manuscript. All authors read and approved the final version of the manuscript.

## Funding

This research was funded by the Deutsche Forschungsgemeinschaft (DFG), grant number Project-ID 251293561 – Collaborative Research Center (CRC) 1149, the German Ministry of Defense, and an unrestricted research grant from the Dr. Franz Köhler Chemie GmbH, Bensheim, Germany. The funder was not involved in the study design, collection, analysis, interpretation of data, the writing of this article, or the decision to submit it for publication. All authors declare no other competing interests.

## Conflict of Interest

The authors declare that the research was conducted in the absence of any commercial or financial relationships that could be construed as a potential conflict of interest.

## Publisher's Note

All claims expressed in this article are solely those of the authors and do not necessarily represent those of their affiliated organizations, or those of the publisher, the editors and the reviewers. Any product that may be evaluated in this article, or claim that may be made by its manufacturer, is not guaranteed or endorsed by the publisher.

## References

[1] Datzmann T Hoffmann A McCook O Merz T Wachter U Preuss J. Effects of sodium thiosulfate (Na_2_S_2_O_3_) during resuscitation from hemorrhagic shock in swine with preexisting atherosclerosis. Pharmacol Res. (2020) 151:104536. 10.1016/j.phrs.2019.104536 31734346

[2] Marutani E Ichinose F: Emerging pharmacological tools to control hydrogen sulfide signaling in critical illness. Intensive Care Med Exp. (2020). 8:5. 10.1186/s40635-020-0296-4 PMC6994583 32006269

[3] Neuwelt EA Gilmer-Knight K Lacy C Nicholson HS Kraemer DF Doolittle ND. Hornig GW,: Toxicity profile of delayed high dose sodium thiosulfate in children treated with carboplatin in conjunction with blood-brain-barrier disruption. Pediatr Blood Cancer. (2006) 47:174–82. 10.1002/pbc.20529 16086410

[4] Marutani E Yamada M Ida T Tokuda K Ikeda K Kai S. Thiosulfate mediates cytoprotective effects of hydrogen sulfide against neuronal ischemia. J Am Heart Assoc. (2015) 4:e002125. 10.1161/JAHA.115.002125 26546573 PMC4845224

[5] Shan H Qiu J Chang P Chu Y Gao C. Wang Het al: Exogenous hydrogen sulfide offers neuroprotection on intracerebral hemorrhage injury through modulating endogenous H[[sb]]2[[/s]]S metabolism in mice. Front Cell Neurosci. (2019) 13:349. 10.3389/fncel.2019.00349 31440142 PMC6693577

[6] Bucci M Vellecco V Cantalupo A Brancaleone V Zhou Z Evangelista S. Hydrogen sulfide accounts for the peripheral vascular effects of zofenopril independently of ACE inhibition. Cardiovasc Res. (2014) 102:138–47. 10.1093/cvr/cvu026 24501330

[7] McCook O Radermacher P Volani C Asfar P Ignatius A Kemmler J. H_2_S during circulatory shock: some unresolved questions. Nitric Oxide. (2014) 41:48–61. 10.1016/j.niox.2014.03.163 24650697 PMC4229245

[8] Merz T Stenzel T Nußbaum B Wepler M Szabo C Wang R. Cardiovascular disease and resuscitated septic shock lead to the downregulation of the H_2_S-producing enzyme cystathionine-γ-lyase in the porcine coronary artery. Intensive Care Med Exp. (2017) 5:17. 10.1186/s40635-017-0131-8 28321823 PMC5359268

[9] Stenzel T Weidgang C Wagner K Wagner F Gröger M Weber S. Association of kidney tissue barrier disrupture and renal dysfunction in resuscitated murine septic shock. Shock. (2016) 46:398–404. 10.1097/SHK.0000000000000599 26926005

[10] Merz T Wepler M Nußbaum B Vogt J Calzia E Wang R. Cystathionine-γ-lyase expression is associated with mitochondrial respiration during sepsis-induced acute kidney injury in swine with atherosclerosis. Intensive Care Med Exp. (2018) 6:43. 10.1186/s40635-018-0208-z 30343340 PMC6195873

[11] Merz T Lukaschewski B Wigger D Rupprecht A Wepler M Gröger M. Interaction of the hydrogen sulfide system with the oxytocin system in the injured mouse heart. Intensive Care Med Exp. (2018) 6:41. 10.1186/s40635-018-0207-0 30341744 PMC6195501

[12] Wigger DC Gröger N Lesse A Krause S Merz T Gündel H. Maternal separation induces long-term alterations in the cardiac oxytocin receptor and cystathionine γ-lyase expression in mice. Oxid Med Cell Longev. (2020) 2020:4309605. 10.1155/2020/4309605 32082478 PMC7007946

[13] Nußbaum BL McCook O Hartmann C Matallo J Wepler M Antonucci E. Left ventricular function during porcine-resuscitated septic shock with pre-existing atherosclerosis. Intensive care Med Exp 4:14, 2016 erratum in: Intensive Care. Med Exp. (2016) 4:18. 10.1186/s40635-016-0092-3 27271248 PMC4894859

[14] Merz T Denoix N Wigger D Waller C Wepler M Vettorazzi S. The role of glucocorticoid receptor and oxytocin receptor in the septic heart in a clinically relevant, resuscitated porcine model with underlying atherosclerosis. Front Endocrinol. (2020) 11:299. 10.3389/fendo.2020.00299 32477273 PMC7239997

[15] Denoix N Merz T Unmuth S Hoffmann A Nespoli E Scheuerle A. Cerebral immunohistochemical characterization of the H_2_S and the oxytocin systems in a porcine model of acute subdural hematoma. Front Neurol. (2020) 11:649. 10.3389/fneur.2020.00649 32754111 PMC7358568

[16] Taccone FS De Backer D. Is cerebral microcirculation really preserved in shock states? Crit Care Med. (2010) 38:1008–9. 10.1097/CCM.0b013e3181d16958 20168171

[17] Nistor M Behringer W Schmidt M Schiffner R. A systematic review of neuroprotective strategies during hypovolemia and hemorrhagic shock. Int J Mol Sci. (2017) 18:2247. 10.3390/ijms18112247 29072635 PMC5713217

[18] Ida KK Otsuki DA Sasaki AT Borges ES Castro LU Sanches TR. Effects of terlipressin as early treatment for protection of brain in a model of haemorrhagic shock. Crit Care. (2015) 19:107. 10.1186/s13054-015-0825-9 25888229 PMC4373118

[19] Jakobsen RP Nielsen TH. Mølstrøm, S, Nordström CH, Granfeldt A, Toft P: Moderately prolonged permissive hypotension results in reversible metabolic perturbation evaluated by intracerebral microdialysis - an experimental animal study. Intensive Care Med Exp. (2019) 7:67. 10.1186/s40635-019-0282-x 31802303 PMC6892994

[20] Ida KK Chisholm KI Malbouisson LMS Papkovsky DB Dyson A Singer M. Protection of cerebral microcirculation, mitochondrial function, and electrocortical activity by small-volume resuscitation with terlipressin in a rat model of haemorrhagic shock. Br J Anaesth. (2018) 120:1245–54. 10.1016/j.bja.2017.11.074 29793592

[21] Lin KH Liu CL Kuo WW Paul CR Chen WK Wen SY. Early fluid resuscitation by lactated Ringer's solution alleviate the cardiac apoptosis in rats with trauma-hemorrhagic shock. PLoS One 11:e0165406, 2016 Erratum for. PLoS ONE. (2016) 11:e0168419. 10.1371/journal.pone.0168419 27780234 PMC5079564

[22] Schiffner R Bischoff SJ Lehmann T Rakers F Rupprecht S Reiche J. Redistribution of cerebral blood flow during severe hypovolemia and reperfusion in a sheep model: critical role of α1-adrenergic signaling. Int J Mol Sci. (2017) 18:1031. 10.3390/ijms18051031 28492488 PMC5454943

[23] Coletti R Almeida-Pereira G Elias LL Antunes-Rodrigues J. Effects of hydrogen sulfide (H_2_S) on water intake and vasopressin and oxytocin secretion induced by fluid deprivation. Horm Behav. (2015) 67:12–20. 10.1016/j.yhbeh.2014.11.008 25436932

[24] Coletti R de Lima JBM Vechiato FMV de Oliveira FL Debarba LK Almeida-Pereira G. Nitric oxide acutely modulates hypothalamic and neurohypophyseal carbon monoxide and hydrogen sulphide production to control vasopressin, oxytocin and atrial natriuretic peptide release in rats. J Neuroendocrinol. (2019) 31:e12686. 10.1111/jne.12686 30633838

[25] Rankin SL Partlow GD McCurdy RD Giles ED Fisher KR. Postnatal neurogenesis in the vasopressin and oxytocin-containing nucleus of the pig hypothalamus. Brain Res. (2003) 971:189–96. 10.1016/S0006-8993(03)02350-3 12706235

[26] Zhu XY Gu H Ni X. Hydrogen sulfide in the endocrine and reproductive systems. Expert Rev Clin Pharmacol. (2011) 4:75–82. 10.1586/ecp.10.125 22115350

[27] Ruginsk SG Mecawi AS da Silva MP Reis WL Coletti R de Lima JB. Gaseous modulators in the control of the hypothalamic neurohypophyseal system. Physiology (Bethesda). (2015) 30:127–38. 10.1152/physiol.00040.2014 25729058

[28] Horn EM Waldrop TG. Oxygen-sensing neurons in the caudal hypothalamus and their role in cardiorespiratory control. Respir Physiol. (1997) 110:219–28. 10.1016/S0034-5687(97)00086-8 9407614

[29] Snijder PM Frenay AR de Boer RA Pasch A Hillebrands JL Leuvenink HG. Exogenous administration of thiosulfate, a donor of hydrogen sulfide, attenuates angiotensin II-induced hypertensive heart disease in rats. Br J Pharmacol. (2015) 172:1494–504. 10.1111/bph.12825 24962324 PMC4369259

[30] Merz T Denoix N Wepler M Gäßler H Messerer DAC Hartmann C. H_2_S in acute lung injury: a therapeutic dead end(?). Intensive Care Med Exp. (2020). 8:33. 10.1186/s40635-020-00324-0 PMC7746418 33336306

[31] Szabo C Papapetropoulos A. Pharmacological modulation of H_2_S levels: H_2_S donors and H_2_S biosynthesis inhibitors. Pharmacol Rev. (2017) 69:497–564. 10.1124/pr.117.014050 28978633 PMC5629631

[32] Sakaguchi M Marutani E Shin HS Chen W Hanaoka K Xian M. Sodium thiosulfate attenuates acute lung injury in mice. Anesthesiology. (2014) 121:1248–57. 10.1097/ALN.0000000000000456 25260144 PMC4237715

[33] Hartmann C Loconte M Antonucci E Holzhauser M Hölle T Katzsch D. Effects of hyperoxia during resuscitation from hemorrhagic shock in swine with preexisting coronary artery disease. Crit Care Med. (2017) 45:e1270–1279. 10.1097/CCM.0000000000002767 29028763

[34] Matějková Š Scheuerle A Wagner F McCook O Matallo J Gröger M. Carbamylated erythropoietin-FC fusion protein and recombinant human erythropoietin during porcine kidney ischemia/reperfusion injury. Intensive Care Med. (2013) 39:497–510. 10.1007/s00134-012-2766-y 23291730

[35] Denoix N McCook O Ecker S Wang R Waller C Radermacher P. The interaction of the endogenous hydrogen sulfide and oxytocin systems in fluid regulation and the cardiovascular system. Antioxidants (Basel). (2020) 9:748. 10.3390/antiox9080748 32823845 PMC7465147

[36] McCook O Scheuerle A Denoix N Kapapa T Radermacher P Merz Merz T: Localization of the hydrogen sulfide and oxytocin systems at the depth of the sulci in a porcine model of acute subdural hematoma. Neural Regen Res. (2021) 16:2376–82. 10.4103/1673-5374.313018 33907009 PMC8374554

[37] Cavaillon JM Annane D. Compartmentalization of the inflammatory response in sepsis and SIRS. J Endotoxin Res. (2006) 12:151–70. 10.1179/096805106X102246 16719987

[38] Neuwelt EA Brummett RE Doolittle ND Muldoon LL Kroll RA Pagel MA. First evidence of otoprotection against carboplatin-induced hearing loss with a two-compartment system in patients with central nervous system malignancy using sodium thiosulfate. J Pharmacol Exp Ther. (1998) 286:77–84. 9655844

[39] Rise IR Kirkeby OJ. Effect of cerebral ischaemia on the cerebrovascular and cardiovascular response to haemorrhage. Acta Neurochir (Wien). (1998) 140:699–706. 10.1007/s007010050165 9781284

[40] Sharma HS Miclescu A Wiklund L. Cardiac arrest-induced regional blood-brain barrier breakdown, edema formation and brain pathology: a light and electron microscopic study on a new model for neurodegeneration and neuroprotection in porcine brain. J Neural Transm. (2011) 118:87–114. 10.1007/s00702-010-0486-4 20963453

[41] Nikolian VC Georgoff PE Pai MP Dennahy IS Chtraklin K Eidy H. Valproic acid decreases brain lesion size and improves neurologic recovery in swine subjected to traumatic brain injury, hemorrhagic shock, and polytrauma. J Trauma Acute Care Surg. (2017) 83:1066–73. 10.1097/TA.0000000000001612 28697014

[42] Bronshvag MM. Cerebral pathophysiology in hemorrhagic shock. Nuclide scan data, fluorescence microscopy, and anatomic correlations. Stroke. (1980) 11:50–9. 10.1161/01.STR.11.1.50 7355430

[43] Meybohm P Gruenewald M Zacharowski KD Albrecht M Lucius R Fösel N. Mild hypothermia alone or in combination with anesthetic post-conditioning reduces expression of inflammatory cytokines in the cerebral cortex of pigs after cardiopulmonary resuscitation. Crit Care. (2010) 14:R21. 10.1186/cc8879 20158893 PMC2875536

